# Alexander Leaf, MD (1920–2012)—A tribute to a life in physiology and medicine

**DOI:** 10.3389/fphys.2013.00006

**Published:** 2013-01-25

**Authors:** George E. Billman

**Affiliations:** Department of Physiology and Cell Biology, The Ohio State UniversityColumbus, OH, USA

**Figure 1:**
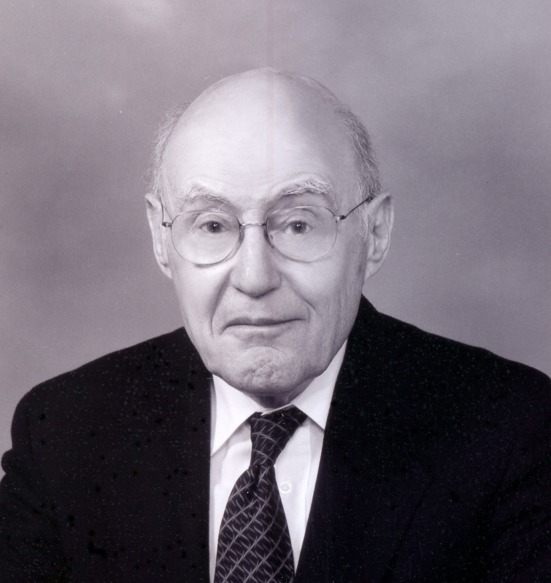
**Photograph of Alexander Leaf, MD (1920–2012)**. Photograph provide by the courtesy of Dr. J. X. Kang.

It is with deep sorrow that I announce the recent passing of Dr. Alexander Leaf (Figure 1), one of the giants in cardio-renal physiology and medicine. After a short illness, Dr. Leaf died peacefully at his home in Concord, MA on December 24, 2012 at the age of 92 years. Those of us who worked with Alex can personally testify to his sense of humor, his humility, his enthusiasm for research, and his tireless quest for scientific truth. He was a supportive mentor to literally hundreds of young scientists and physicians—a legacy that will continue to bear fruit for many years to come. Alex had a long and distinguished career that is difficult to summarize adequately in the short space allotted for this memorial. I encourage all the readers of this brief tribute to read an autobiography that Alex wrote a little over 10 years ago as the introductory chapter of the 2001 *Annual Review of Physiology* (Leaf, 2001). His essay provides an excellent overview of his accomplishments and clearly conveys his sense of humor and humility. Alex was the definitive gentleman and scholar.

In the remainder of the present essay, I shall attempt to provide the reader with a sense of Alex, the man, as well as a limited summary of his remarkable accomplishments as a scientist and physician. Alex was born in Yokohama, Japan April 10, 1920 where his parents had escaped from Czarist Russia shortly before the Bolshevik revolution. They left Japan and settled in Seattle in 1922, where his father opened a dental practice. I recall him fondly describing an idyllic boyhood, hiking and exploring the still largely virgin forests of the Pacific Northwest. Alex claims that he was not the best student, but I think this is an example of his humility as he was awarded a partial scholarship to Harvard University. However, as it was the height of the depression, he opted to live at home and complete his undergraduate education at the much more affordable University of Washington (tuition $30/semester), graduating with a degree in Chemistry in 1940. Upon graduation he enrolled in the medical program at the University of Michigan. With the winds of war approaching gale force, he enlisted in the Army Medical Corps and, due to an accelerated program, obtained his medical degree in 3 years, graduating in 1943. The army allowed him to complete residency programs at Massachusetts General Hospital (MGH) and the Mayo Clinic before assuming active duty. Although he had requested an overseas assignment, he completed his military obligation at Beaumont General Hospital in El Paso, Texas.

After his release from the military in 1946, he returned to the University of Michigan to assist his mentor, Prof. L. H. Newburgh, in his research. He spent the next 2 years investigating water and electrolyte metabolism. As Dr. Newburgh was soon to retire, he advised Alex to accept a position at Massachusetts General. Alex arrived in Boston with a Rockefeller fellowship that allowed him to establish an independent laboratory. In the 1950's Alex performed seminal studies that established that the higher osmolality of the intracellular environment was maintained not by the active transport of water (the prevalent view at the time) but rather due to the passive redistribution of water secondary to the transport of sodium and chloride into the extracellular space, thereby regulating cell volume (Leaf, 1956, 1959; Maffly and Leaf, 1959). He also completed studies that evaluated neurohormonal regulation of water and electrolyte balance (Leaf et al., 1953) and, during a sabbatical leave, completed studies with Hans H. Ussing in Copenhagen and Hans Krebs at Oxford University. Having mastered a new technique, the Ussing chamber (Ussing and Zerhan, 1951), to study electrolyte flux across the toad urinary bladder, Alex continued to climb the academic ladder, becoming the Jackson Professor of Clinical Medicine and the Chair of the Department of Clinical Medicine at MGH/Harvard in 1966, a position he held until 1981. Five students who completed their training while Alex was the Chair of Clinical Medicine have been awarded the Nobel prize, and many more have become leading figures in science and medicine both within and outside of the United States.

Alex indicates in his autobiography that he became increasing dissatisfied with what he saw in the clinic. His extensive travels in remote regions of the globe for the World Health Organization convinced him that many of the debilitating diseases seen by physicians in Western society were the result of lifestyle choices and could be prevented. In his words: “We physicians waited in our offices until a patient came to us with their disease well advanced. There was very little we could do to cure them of these chronic ailments. Increasingly we responded with ingenious and expensive technology that mostly provided palliative relief, at best” (Leaf, 2001). In 1981, he resigned as Chief of Clinical Medicine to form and to chair of a new department of preventive medicine and epidemiology, a position he held until his mandatory retirement at age 70 years in 1990. Upon retirement and now freed from administrative burdens, Alex began a new career focusing on the role of diet (and, to a lesser degree, exercise) in the prevention of disease. He is perhaps best known for his pioneering work on the cardiovascular effects of omega-3 fatty acids/fish oils in the prevention of cardiovascular disease. I had the great good fortune to participate in these studies shortly after Alex's retirement when he generously invited me to evaluate the effects of omega-3 fatty acids in a canine model of sudden cardiac death (Schwartz et al., 1984; Billman, 2006). We confirmed Alex's early findings on the salutatory properties on isolated neonatal rat cardiomyocytes (Hallaq et al., 1990) in this conscious animal model. Specifically, we demonstrated that acute intravenous administration of an emulsion of either fish oil or purified omega-3 fatty acids could prevent ischemically-induced ventricular fibrillation (Billman et al., 1994, 1997, 1999). Working with Dr. Jing Kang, Alex built upon these earlier studies, documenting that these fatty acids reduced myocyte membrane excitability via inhibitory actions on various ion channels (particularly sodium and L-type calcium channels) (Kang et al., 1995; Xiao et al., 1995, 1997; Kang and Leaf, 1996).

During his illustrious career Alex published in excess of 340 full length manuscripts that have been cited nearly 20,000 times. He was the recipient of numerous awards and honors including election as a Fellow to the very prestigious National Academy of Sciences (USA) in 1972 and to the Institute of Medicine, 1978. Alex is survived by his wife Barbara, whom he married in 1943, three daughters (Caroline, Rebecca, and Tamara), and two grandchildren (Alexander and Anna Norregaard). He is also survived by his many students, colleagues, and their students—his intellectual children and grandchildren. Readers are encouraged to post the comments and reminiscences using the comment link that accompanies this memorial.
